# Effect of Soaking Treatments in Acid and Salt Solutions on Physicochemical, Structural, Thermal and Rheological Properties of Porang (*Amorphophallus muelleri* Blume) Flour

**DOI:** 10.17113/ftb.62.04.24.8503

**Published:** 2024-12

**Authors:** Lia Ratnawati, Novita Indrianti, Nok Afifah, Riyanti Ekafitri, Enny Sholichah, Dewi Desnilasari, Woro Setiaboma, Dita Kristanti, Achmat Sarifudin

**Affiliations:** 1 Research Center for Appropriate Technology-National Research and Innovation Agency Jl. K.S. Tubun No. 5 Subang 41213, West Java, Indonesia; 2 Research Center for Applied Microbiology-National Research and Innovation Agency Jl. Raya Jakarta-Bogor KM. 46, Cibinong-Bogor, West Java, Indonesia; 3 Research Center of Food Processing and Technology-National Research and Innovation Agency Jl. Raya Jogja-Wonosari, Playen, Yogyakarta, Indonesia

**Keywords:** *Amorphophallus muelleri* Blume, porang flour, soaking in acid solution, salt solution

## Abstract

**Research background:**

Porang (*Amorphophallus muelleri* Blume) contains a high amount of starch, glucomannan and Ca-oxalate. Soaking porang tuber in acid (citric acid) and salt (sodium chloride) solutions affects the Ca-oxalate content, functional, rheological and thermal properties of porang flour. The aim of this study is to thoroughly investigate the effect of soaking treatments in acid and salt solutions at different temperatures on the physicochemical, rheological and thermal properties, functional groups, molecular mass and morphology of porang flour.

**Experimental approach:**

Soaking treatments in acid and salt solutions at different temperatures affect the properties of porang flour. This research investigated the effect of soaking porang slices in citric acid (5 %) and sodium chloride (8 %) solutions at different soaking temperatures (25, 55 and 85 °C for 1 h) on the properties of the resulting porang flour.

**Results and conclusions:**

The results of this study showed that all treatments successfully reduced the Ca-oxalate mass fraction in porang flour. The acid treatments reduced the glucomannan content more than the salt treatments. Soaking in acid solutions also decreased viscosity, molecular mass and thermal stability of porang flour. When the soaking temperature was increased, the Ca-oxalate mass fraction, molecular mass, viscosity and thermal stability of the porang flour decreased. The acid and salt treatments did not change the FTIR patterns. The morphological analysis showed that the acid and salt treatments resulted in particles with rough surface and short crystal needles of Ca-oxalate. Soaking the porang slices in salt solution at a temperature of 55 °C was the most effective treatment to reduce the Ca-oxalate content of porang flour while retaining its other quality parameters.

**Novelty and scientific contribution:**

The results of this study provided a comprehensive understanding of the effect of soaking porang slices in acid and salt solutions on the properties of the obtained porang flour. They can be used as scientific evidence on how to treat the porang slices to obtain the best quality of porang flour.

## INTRODUCTION

Porang has become one of the most widely grown tuber crops in Indonesia in the last five years. It contains a high amount of carbohydrate groups, such as starch and glucomannan. Yanuriati *et al.* (*1*) reported that porang flour contains 79.91 % glucomannan. Glucomannan is a polysaccharide molecule containing d-glucose and d-mannose, which are built by 1-4-glycoside bonds with an acetyl group at the C-6 position. Glucomannan is also categorised as a dietary fibre that can reduce the risk of haemorrhoids, prevent small pouches in the colon (diverticular disease), regulate cholesterol levels and also help control blood sugar levels. It is a food additive that is often used as a thickener, texture-forming or gelling agent, fat replacer and stabiliser (*2*, *3*). Therefore, porang flour has the potential to be developed into a functional food ingredient.

In addition to glucomannan, porang flour also contains an undesirable compound, Ca-oxalate, which limits its use as a food ingredient. Ca-oxalate is typically found in porang tubers, slices and flour at around 15.98, 10.38 and 1.98 %, respectively (*4*–*6*). If consumed in large quantities, Ca-oxalate can cause health problems such as itching, a burning sensation in the stomach and irritation when it comes in contact with the skin, mouth and digestive tract. Therefore, efforts must be made to reduce the Ca-oxalate content in porang flour so that it can be safely used as a food ingredient.

Previous studies reported that a combined treatment of soaking in salt solution and heating reduced the Ca-oxalate content more effectively than either soaking in salt solution or heating alone (*2*). The effect of soaking of different types of tuber crops (cocoyam, *Dioscorea dumetorum*, taro, wild bitter yam, anchote, sweet potato and potato) in different solutions (potassium disulfite, sodium disulfite, corn infusion and tamarind infusion) and at different temperatures (70–100 °C) on the functional properties of their flours was also investigated (*7*–*11*). Treatment with salt and citric acid can increase the water absorption capacity of tuber flour (*9*). Adejumo and Asema (*7*) and Melese and Keyata (*9*) found that soaking temperature affects the water absorption capacity, oil absorption capacity, swelling power and viscosity of tuber flour. Based on previous studies, soaking with salt and acid affects both the Ca-oxalate content and the functional properties of tuber flour, which needs to be further investigated.

Besides affecting the Ca-oxalate content and functional properties, the addition of acid or salt also changes the rheological and thermal properties of polysaccharides (*12*, *13*). However, the effects of this treatment on the physicochemical, rheological and thermal properties of porang flour have not yet been investigated. Therefore, the aim of this study is to thoroughly investigate the properties of porang flour treated by soaking in acid and salt solutions at different temperatures. More specifically, the Ca-oxalate, ash, starch, amylose and glucomannan content, colour (L*, a*, b* and Δ*E*), rheological and thermal properties, functional groups, molecular mass and morphology of porang flour were also investigated in this study.

## MATERIALS AND METHODS

### Materials

Porang tubers were obtained from a local farmer in Subang, West Java, Indonesia. Acid (citric acid) and salt (sodium chloride) for domestic use were obtained from a local market (Jakarta, Indonesia). Analytical-grade chemicals were used for the analysis.

### Sample preparation

Porang flour was prepared according to Sarifudin *et al.* (*14*). Porang tubers were washed, peeled, sliced and soaked (*m*(porang):*V*(solution)=1:2) in a solution of 5 % citric acid (A) and 8 % sodium chloride (S) at temperatures of 25, 55 and 85 °C for 1 h. To regulate the temperature accurately, a calibrated thermometer was used to monitor and adjust the heating setting of the stove (RI-522C; Rinnai, Jakarta, Indonesia) and to ensure consistent thermal equilibrium in the system. The control sample (C) was prepared in the same steps, but without soaking in any solutions. The samples were rinsed three times under running water, dried in a cabinet dryer (type 090; Jenn Chiang Machinery Works Co., Ltd, Feng Yuan, Taiwan) at 50 °C for 12 h, ground in a grinder (HR2115; Philips, Batam, Indonesia) and sieved through 40 mesh. All samples were prepared in triplicate. The sample codes were C for the control/untreated porang flour; A25, A55 and A85 for the samples soaked in citric acid at 25, 55 and 85 °C, respectively; and S25, S55 and S85 for the samples soaked in sodium chloride at 25, 55 and 85 °C, respectively.

### Determination of physicochemical properties

Ca-oxalate was determined according to the titration method (*15*) with some modifications (in the dilution factor). The sample (1 g) was placed in a 250-mL Erlenmeyer flask and mixed with 5 mL HCl (*c*=6 M) and 95 mL of distilled water. It was then heated in a hotplate stirrer (SMHS-3; Daihan Scientific, Gangwon-do, South Korea) at 100 °C for 1 h. As much as 125 mL distilled water was added and mixed well with the sample. After that, the sample was filtered through filter paper (Whatman No. 40). A volume of 30 mL of the filtrate was then mixed with 120 mL of distilled water and 10 mL of 20 % H_2_SO_4_. Next, it was heated at 85 °C for 5 min with a shaking water bath (1086; Gesellschaft für Labortechnik (GFL), Burgwedel, Germany). The sample was then immediately titrated with standardised KMnO_4_ (*c*=0.05 M) until a faint pink colour persisted for 30 s. The Ca-oxalate content (in %) was calculated using the following equation:


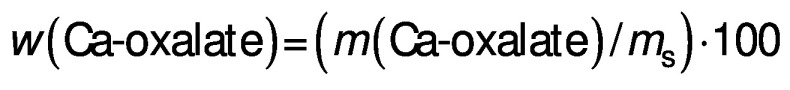
 /1/

where *m*_s_ is the mass of the sample (g).

The ash content was determined according to AOAC method 923.03 (*16*). The starch content was determined by direct acid hydrolysis and titration (*17*). A sample of 0.5 g was placed in a 100-mL glass beaker, 50 mL of distilled water were added, the mixture was stirred at room temperature for 1 h and separated using a centrifuge (SL 40R; Thermo Scientific, Osterode am Harz, Germany) at 1917×*g* for 15 min. The filtrate was then discarded and 50 mL of distilled water were added to the sample and stirred for 1 h. The suspension was filtered and washed with distilled water to obtain a filtrate volume of 250 mL. A volume of 200 mL of distilled water and 20 mL of 20 % HCl were added to the residue. The mixture was refluxed for 2.5 h, cooled and neutralised with 30 % NaOH and then 3 % CH_3_COOH were added. The solution was transferred to a 500-mL volumetric flask, distilled water was added, the solution was filtered and 25 mL of the filtrate were taken to which 25 mL of Luff-Schoorl solution were added. The mixture was heated for 10 min, cooled and 10 mL of 20 % KI and 25 mL of 25 % H_2_SO_4_ were added. The solution was titrated with 0.1 M Na_2_S_2_O_3_ until it turned pale yellow. As much as 1 % of starch was added until the solution turned purplish-blue. The titration was continued until the purplish blue colour disappeared. The amount of 0.1 M Na_2_S_2_O_3_ was calculated. Blank solution was tested by replacing the filtrate with 25 mL distilled water. Starch mass fraction (in %) was calculated using the following equation:


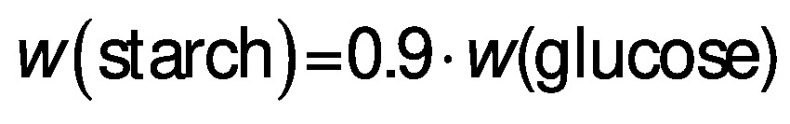
 /2/

The amylose content was analysed with a spectrophotometer (UV-Vis 1900; Shimadzu Corporation, Kyoto, Japan) at 620 nm (*18*). A mass of 100 mg was weighed in a test tube and 1 mL of 95 % ethanol and 9 mL of 1 M NaOH were added. The solution was heated in a water bath at 100 °C for 10 min and cooled for 1 h. The sample was then transferred to a 100-mL volumetric flask and diluted to a mark with distilled water. After dilution, 5 mL of the solution were transferred to a 100-mL volumetric flask and 1 mL of 1 M CH_3_COOH and 2 mL of 2 % I_2_ were added. The solution was then diluted with distilled water until it reached the volume of 100 mL. It was shaken and allowed to stand for 20 min. Then the absorbance was measured with a spectrophotometer (UV-Vis 1900; Shimadzu Corporation) at *λ*=620 nm. Potato amylose was used as a standard. The amylose mass fraction (in %) was calculated using the following equation:


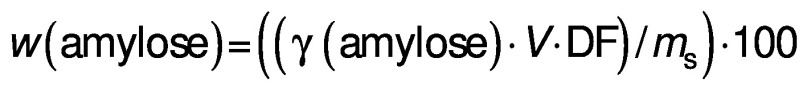
 /3/

where *γ*(amylose) is the amylose concentration (mg/mL), *V* is the total volume (mL), DF is the dilution factor and *m*_s_ is the sample mass (mg).

The glucomannan content of the sample was analysed according to a method by Peiying *et al*. (*19*) with slight modifications (stirring time). The sample (0.2 g) was added to 50 mL of formic acid-NaOH buffer (pH=3.04) and stirred for 15 h at room temperature. The mixture was then diluted to 100 mL with formic acid-NaOH buffer in a volumetric flask, followed by centrifugation (2504×*g*, 40 min, SL 40R; Thermo Scientific). The extract (2 mL) was pipetted into a 10-mL volumetric flask and 1 mL H_2_SO_4_ (*c*=3 M) was added. After that, the solution was mixed in a vortex mixer (VM-300; Gemmy Industrial Corp., Taipei, Taiwan) and then heated for 90 min at 100 °C in a shaking water bath. The sample was allowed to cool to room temperature before the addition of 1 mL NaOH (*c*=6 M). The solution was then made up to 10 mL with distilled water to form the glucomannan hydrolysate. The glucomannan extract and hydrolysate were subjected to colourimetric reactions and distilled water was used as a blank. About 0.6 mL of 3,5-dinitrosalicylic acid solution was added to 0.8 mL of the sample. Each mixture was heated for 5 min in a boiling water bath and cooled to room temperature before being diluted to 10 mL with distilled water in a volumetric flask. Absorbance was then measured at *λ*=540 nm using a UV-Vis spectrophotometer (UV-Vis 1900; Shimadzu Corporation). The glucomannan mass fraction (in %) was calculated using the following equation:


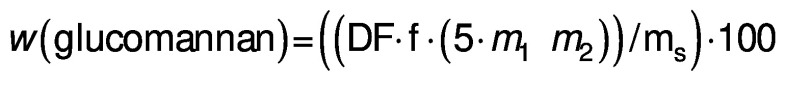
 /4/

where DF is the dilution factor, f is the correction factor, *m*_1_ is the glucose mass in the hydrolysate (mg), *m*_2_ is the glucose mass in the extract (mg) and *m*_s_ is the mass of sample (mg).

Solubility was determined according to Yanuriati *et al*. (*1*). The colour was assessed with a chromameter (NH310; 3NH Technology, Guangdong, PR China). The colour parameters including L* (lightness), a* (redness), b* (yellowness) and Δ*E* (total colour difference) were reported.

### Fourier transform infrared measurement

The powder of the sample was placed into the Fourier transform infrared (FTIR) sample compartment. FTIR measurements were carried out using a FTIR (Alpha II; Bruker, Ettlingen, Germany) at a resolution of 4 cm^-1^ in the range of 400-4000 cm^-1^.

### Molecular mass measurement

The molecular mass distribution was measured using the gel permeation chromatography (GPC) system (1260 Infinity II; Agilent Technologies, Waldbronn, Germany) with multiple detectors, namely a viscometer and a refractive index detector (Agilent Technologies, Church Stretton, UK). The chromatographic column used PL aquagel-OH mixed-H 8 µm 300 mm×7.5 mm (Agilent Technologies, Church Stretton) and the system was calibrated with polyethylene oxide/polyethylene glycol (ReadyCal-Kit PEO/PEG; Agilent, Mainz, Germany), which has a measurement range of *M*=10^2^–1.2·10^6^ g/mol. The mobile phase was a water solution containing 0.02 % NaN_3_. The sample was dissolved in water for chromatography (1 mg/mL), stirred for 30 min, centrifuged (SL 40R; Themo Scientific) at 2504×*g* for 15 min and filtered using Millipore 0.45 μm filter (Agilent Captiva Econofilter; Agilent Technologies, Bejing, PR China). A volume of 20 µL of sample solution was injected at a constant flow rate of 0.5 mL/min and the columns and detectors were maintained at 35 °C (*20*). Molecular mass measurement provides the data of *M*_n_ (number average molecular mass), *M*_m_ (mass average molecular mass) and polydispersity index (PDI). *M*_n_, *M*_m_ and PDI were calculated according to the following equations:


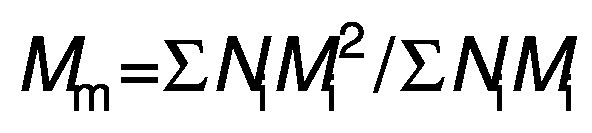
 /5/


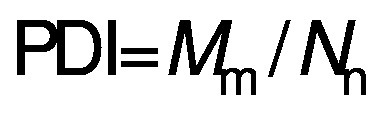
 /6/

where *M*_i_ is the molecular mass of a chain and *N*_i_ is the number of chains of that molecular mass.


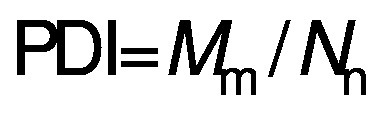
 /7/

### Thermal measurement

The thermal properties were analysed using a derivative thermogravimetry (DTG-60; Shimadzu). An empty aluminium pan was used as a reference. A mass of 5 mg of the sample was weighed into the pan. The measurement was conducted using nitrogen gas at a flow rate of 100 mL/min and a heating rate of 10 °C/min within a temperature range of 25 to 450 °C. Thermal measurement provides mass loss, onset temperature (*t*_o_), endset temperature (*t*_c_), peak temperature (*t*_p_) and enthalpy change (Δ*H*).

### Rheological measurement

The apparent viscosity was measured according to Yanuriati *et al*. (*1*). A solution of porang flour of 3 % (*m*/*m*) was prepared. The steady shear flow of samples was measured using a rheometer (HAAKE Mars 40; Thermo Scientific, Karsluhe, Germany) at a constant temperature of 25 °C and a shear rate from 0 to 300 s^−1^. The measurement was carried out using a cone and a gap distance of 4 mm (*21*).

### Morphology

The morphology of the samples was determined using a scanning electron microscope (SEM) (JSM-IT300; JEOL, Tokyo, Japan) which was operated at 20 kV and magnification of 10 000×. The samples were placed on a metal stub and coated with gold.

### Statistical analysis

This study used a completely randomised design to evaluate the effect of acid and salt soaking treatments on the properties of porang flour. Each treatment was repeated three times and physicochemical properties were measured twice. Data from the treated samples were analysed using analysis of variance (ANOVA) at a 95 % confidence interval. If there was a significant difference, the Duncan’s multiple range test (α=5 %) was conducted. In addition, the data from the treated samples were statistically compared with the data from the control samples (without soaking) using Dunnett's test. All data were processed using the SPSS software v. 26 (*22*).

## RESULTS AND DISCUSSION

### Physicochemical properties

The physicochemical properties of porang flour are shown in Table 1. The Ca-oxalate mass fraction ranged from 0.68 to 2.26 %, which confirmed its reduction by up to 70 %. The results indicated that soaking in acid and salt solutions at any temperature significantly reduced the Ca-oxalate content in porang flour. Moreover, soaking porang slices in salt solution at 85 °C resulted in porang flour with the lowest Ca-oxalate (0.68 %). The decrease in Ca-oxalate mass fraction of porang slices can be due to the increased solubility of Ca-oxalate during soaking in acid or salt solutions. Agustin *et al*. (*23*) found that low pH solution has the ability to partially alter water-insoluble Ca-oxalate to water-soluble oxalic acid. In addition, an ion exchange mechanism may occur during soaking Ca-oxalate in salt solution, producing water-soluble sodium oxalate, which is discarded after soaking (*2*). In addition, Toshima (*24*) found that the solubility of Ca-oxalate in water increases with the increase in temperature.

**Table 1:** Physicochemical properties on dry mass basis of porang flour obtained by soaking in acid and salt solutions

Treatment	Parameter									
	*w*(Ca-oxalate)/%	*w*(ash)/%	*w*(starch)/%	*w*(amylose)/%	*w*(glucomannan)/%	solubility/%	L*	a*	b*	Δ*E*
C	(2.26±0.05)	(6.26±0.00)	(25.3±0.6)	(2.9±0.2)	(59.3±2.7)	(40.8±1.8)	(78.1±0.2)	(4.2±0.1)	(12.40±0.04)	(0.00±0.00)
A25	(1.76±0.03)^f^*	(5.4±0.2)^b^*	(22.1±0.8)^e^*	(2.9±0.3)^a^	(53.1±1.2)^b^*	(25.0±1.3)^a^*	(80.4±0.7)^c^	(4.9±0.5)^b^	(13.5±1.1)^b^	(4.46±0.04)^ab^
A55	(1.58±0.04)^e^*	(3.16±0.06)^a^*	(17.02±0.09)^d^*	(3.16±0.09)^a^	(49.3±0.9)^a^*	(38.3±0.8)^c^	(80.0±0.4)^c^	(5.78±0.09)^cd^*	(16.2±1.2)^c^*	(13.0±2.2)^d^
A85	(1.47±0.05)^d^*	(3.30±0.05)^a^*	(16.1±0.6)^cd^*	(4.8±0.3)^d^*	(48.7±0.7)^a^*	(46.7±3.2)^e^*	(79.26±0.01)^c^	(6.0±0.4)^d^*	(16.3±0.3)^c^*	(10.1±0.9)^bcd^
S25	(1.34±0.02)^c^*	(8.2±0.1)^e^*	(15.2±1.2)^c^*	(4.2±0.1)^c^*	(59.4±0.3)^c^	(30.3±1.6)^b^*	(74.6±1.2)^a^*	(5.5±0.5)^bcd^*	(10.6±0.7)^a^*	(7.0±1.7)^bc^
S55	(1.04±0.07)^b^*	(7.92±0.02)^d^*	(13.0±0.7)^b^*	(3.93±0.01)^bc^*	(60.0±1.0)^c^	(33.3±1.0)^b^*	(75.47±3.03)^ab^	(5.1±0.5)^bc^*	(11.2±0.1)^a^	(11.5±3.4)^cd^
S85	(0.68±0.03)^a^*	(6.76±0.08)^c^*	(9.4±0.7)^a^*	(3.7±0.1)^b^*	(58.2±3.3)^bc^	(43.1±1.6)^d^	(77.8±1.4)^bc^	(3.3±0.1)^a*^	(13.6±1.2)^b^	(1.41±0.04)^a^

Results are average value±standard deviation, *N*=3. Different letters in superscript in each column indicate that the values are statistically significantly different (p<0.05). *Statistically significant difference between the treated samples and the untreated sample according to Dunnett's test at p<0.05. C=control/untreated porang flour, A25, A55 and A85=soaked in 5 % citric acid at 25, 55 and 85 °C, respectively, S25, S55 and S85=soaked in 8 % sodium chloride at 25, 55 and 85 °C, respectively

The ash mass fraction of the samples ranged from 3.16 to 8.2 %. Overall, the soaking treatments affected the ash content (p<0.05). Acid treatment significantly reduced the ash mass fraction. The findings of this study are consistent with earlier research by Trithavisup and Charoenrein (*25*), who reported that rice flour soaked in a solution of citric acid and ascorbic acid showed significant decreases in its protein, fat and ash content. The loss of some molecules such as chemical or mineral compounds that were dissolved in the water or acid solutions during treatment may have contributed to the decrease in the ash mass fraction. On the other hand, salt treatment tended to increase the ash content. This might be due to the salt that remained on the surface of the porang slices during soaking. NaCl reacted and bonded with cell wall components such as pectin, protein and fat. Therefore, the salt residues contributed to the high ash content of the porang flour produced by the salt treatment. The results also showed that porang flour tended to have a lower ash content when soaked at a higher temperature than at a low temperature (Table 1). This could be due to the higher temperature assisting the salt leaching from the sample surface during soaking. Ayele *et al*. (*26*) reported that cooking at high temperatures reduced the ash content of yam and taro tubers.

Glucomannan is a desirable component of the porang tuber, while other components are considered impurities, including ash and starch. Table 1 shows that the glucomannan mass fraction of the samples ranged from 48.7 to 53.1 % and 58.2 to 60.0 % in the samples soaked in acid and salt, respectively. The results show that the samples treated with acid had a lower glucomannan mass fraction than the control sample. Soaking in acid may cause degradation of the polysaccharide chains, which results in the release of short fragments of glucomannan chains into the medium, thereby decreasing the glucomannan content of acid-treated samples (*27*). The results also showed that a higher soaking temperature leads to a lower glucomannan mass fraction of the acid-treated samples (Table 1). This result could be due to more intense hydrolysis at higher temperatures, so glucomannan molecules in the sample matrix diffused into the soaking medium. Higher temperatures improve the solubility of polysaccharides and increase their diffusion coefficient (*27*, *28*). As a result, polysaccharide molecules can more easily escape from the entrapped matrix at high temperatures (*28*).

Table 1 shows that the starch and amylose mass fraction of porang flour from the treatments ranged from 9.4 to 25.3 % and from 2.9 to 4.8 %. The results showed that all treatments significantly reduced the starch content and increased the amylose content of porang flour. Acids have the ability to hydrolyse glycosidic bonds by a rapid attack in the amorphous region and the slower attack in the amylopectin fraction in the crystalline region (*29*). As a result, the starch in the porang slices was released into the solution, reducing the starch content. Acid hydrolysis has the ability to break the glycosidic bonds along the polysaccharide chains, including amylose and amylopectin chains, increasing the amylose content. Soaking in salt leads to a loss of starch because the presence of salt can accelerate the breakdown of starch by interacting directly with the starch granules or indirectly by speeding up the caramelisation, which leads to acidity and destruction of granules (*12*). Salt increases the amylose content of porang flour because it is able to dissociate and generate energy, which causes the amylose to break down and form a considerable amount of short amylose (*30*).

The solubility of the treated porang flour samples ranged from 25.0 to 46.7 %. The results showed that the porang flour treated with acid had a higher solubility value than the samples treated with salt. The soaking in acid could cause hydrolysis of polymer chains of glucomannan and starch (*21*, *31*) in the matrix of porang flour. Acid treatment also causes a decrease in the molecular mass of complex carbohydrates and increases the solubility (*32*). Moreover, increasing temperature causes a higher hydrolysis reaction rate (*33*). Therefore, the higher soaking temperature in acid and salt solution increased the solubility of porang flour (Table 1). Since heating weakens the structure of hydrogen bonds and allows water to seep through the insoluble starch, some of the starch breaks down and becomes soluble. Because the starch granules have a high amylopectin content, the hydrogen bonds can be easily broken, increasing the solubility values (*34*).

The effect of immersing porang slices in acid and salt solutions on their colour is shown in Table 1. The colour parameter showed that soaking the porang slices in acid solutions resulted in porang flour with a lighter colour than the control samples. This could be due to the ability of citric acid to minimise the browning effects that occur when sliced tubers are exposed to air (*35*). Citric acid acts as a chelating agent and binds all metals that accelerate browning reactions (*36*). Soaking porang slices in a salt solution reduced the lightness of the porang flour compared with the control samples. This result is consistent with the study by Moreau *et al.* (*12*). In addition, the results showed that soaking porang slices at higher temperatures resulted in porang flour with a lighter colour than soaking at room temperature (Table 1). It is possible that the browning enzymes present in the porang tuber are inactivated when the porang slices are soaked at high temperature (*37*). Therefore, porang flour produced at high soaking temperatures tended to have a light colour, especially sample S85. The S85 sample also had low Δ*E*, indicating that the colour characteristics of the porang flour after the treatment are is similar to the control. The Δ*E* is calculated by comparing the difference between the L*, a* and b* values of the treated sample and the control.

### Fourier transform infrared spectroscopy

**Fig. 1:**
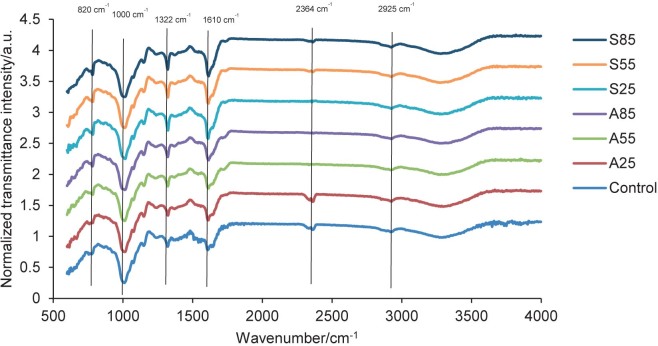
IR patterns of porang flour obtained by soaking in acid and salt solutions. C=control/untreated porang flour, A25, A55 and A85=soaked in 5 % citric acid at 25, 55 and 85 °C, respectively, S25, S55 and S85=soaked in 8 % sodium chloride at 25, 55 and 85 °C

FTIR is used to analyse both organic and inorganic substances. It serves for qualitative evaluation, by recognising functional groups through the presence of prominent peaks and their arrangement, and for quantitative evaluation, by measuring the compound absorption intensity at specific wavenumbers. FTIR can be used to analyse Ca-oxalate compounds in porang flour. Fig. 1 shows the spectra of porang flour at wavenumbers 400-4000 cm^-1^. Acid, salt and temperature treatments did not change the FTIR spectrum pattern of porang flour, but they changed the intensity of several functional groups. The FTIR spectrum pattern of porang flour from this study was similar to that of konjac glucomannan flour according to Wang *et al.* (*38*). Porang flour had a broadband at a wavenumber of 3000-3700 cm^-1^, which indicates the presence of O-H stretching of glucose and mannose glucomannan. N–H stretching was also observed between 3000 and 3700 cm^-1^, indicating proteins (*39*, *40*).The peak observed at approx. 1000 cm^-1^ corresponds to the stretching vibration of the C-O-C bond, which is consistent with the results reported by Azhar *et al.* (*41*). Porang flour also showed a peak at a wavenumber of 2925 cm^-1^, which indicates a symmetric and asymmetric stretching vibration of the alkane group. A strong peak at 1600-1700 cm^-1^ indicates the presence of a carbonyl group (C=O) corresponding to C=O from oxalate and glucomannan (*39*, *42*). The peak at a wavenumber of 820 cm^-1^ showed the characteristics of a mannose peak. The peak at a wavenumber around 2300 cm^-1^ indicated O=C=O stretching (*42*).

### Molecular mass distribution

**Fig. 2:**
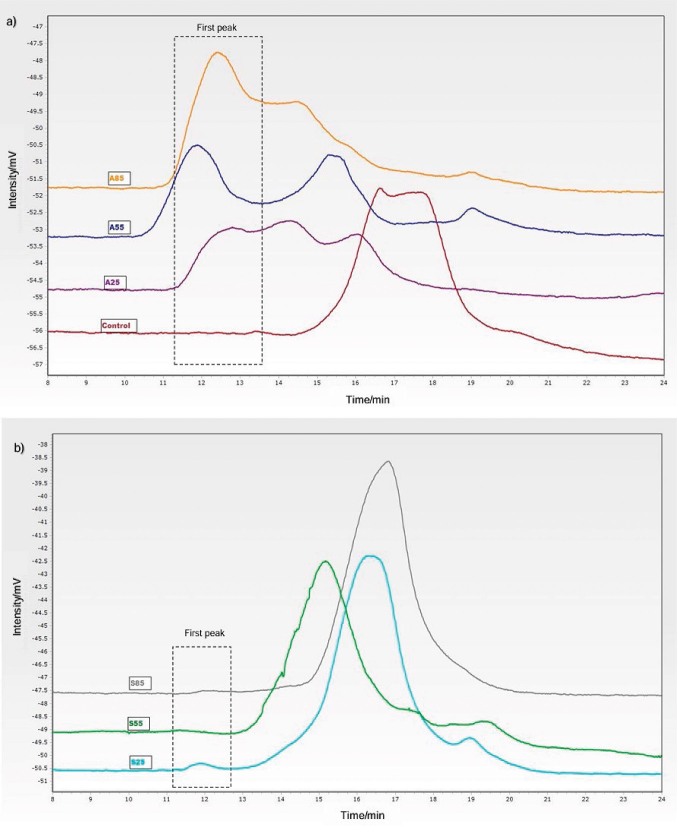
Gel permeation chromatograms of: a) control and acid treatment, and b) salt treatment. C=control/untreated porang flour, A25, A55 and A85=soaked in 5 % citric acid at 25, 55 and 85 °C, respectively, S25, S55 and S85=soaked in 8 % sodium chloride at 25, 55 and 85 °C, respectively

The gel permeation chromatography (GPC) chromatograms of the untreated and treated porang flour are shown in Fig. 2, and the molecular properties associated with the GPC profile are shown in Table 2. The GPC chromatograms (Fig. 2) show that the control and treated samples had several peaks. The samples used for GPC were dissolved in water and therefore contained only certain sizes of polymers, oligomers and monomers. The peak at about 12–13 min represented the glucomannan polymer peak and the next peaks represented monomers and low-molecular-mass oligomers. The chromatograms showed that the control samples and the samples treated with salt contained high-molecular-mass polymers and small amounts of monomers or low-molecular-mass oligomers. This result is consistent with a previous study showing that hydrolysis of glucomannan produces a group of oligosaccharide peaks representing smaller molecules, such as reducing sugars (*43*). This indicated that the salt treatment converted only a small amount of glucomannan polymers into shorter oligomers. However, the acid-treated samples had a chromatogram in which the peaks for the high-molecular-mass polymers and several sizes of oligomers appeared in a relatively larger amount. This was thought to be due to the degradation of the glucomannan polymer chain. These results led to better homogeneity in the salt-treated samples than in acid-treated samples, which was consistent with their *M*_n_ and polydispersity index (PDI) values. The peaks also increased with an increase in soaking temperature, resulting in a higher PDI.

**Table 2:** Molecular mass distribution in porang flour obtained by soaking in acid and salt solutions

**Treatment**	Molecular mass distribution		
	*M*_n_/(g/mol)	*M*_m_/(g/mol)	PDI
**C**	(1010000±76100)	(751000±25700)	(1.35±0.15)
**A25**	(1180000±162000)^a^*	(959000±76700)^b^*	(1.23±0.07)^a^*
**A55**	(1530000±140000)^b^	(1230000±38000)^c^	(1.24±0.08)^a^*
**A85**	(1070000±648000)^a^*	(804000±88900)^a^*	(1.34±0.07)^a^*
**S25**	(1560000±106000)^b^	(1260000±384000)^c^	(1.24±0.09)^a^*
**S55**	(1790000±165000)^b^	(1550000±27100)^d^	(1.16±0.09)^a^*
**S85**	(932000±144000)^a^*	(790000±77600)^a^*	(1.18±0.07)^a^*

Results are average value±standard deviation, *N*=2. Different letters in superscript in each column indicate that the values are statistically significantly different (p<0.05). *Statistically significant difference between the treated samples and the untreated sample according to Dunnett's test at p<0.05. C=control/untreated porang flour, A25, A55 and A85=soaked in 5 % citric acid at 25, 55 and 85 °C, respectively, S25, S55 and S85=soaked in 8 % sodium chloride at 25, 55 and 85 °C, respectively. PDI=polydispersity index

Table 2 shows the molecular mass distribution of the first peak of the samples. The number of average molecular mass (*M*_n_) and the mass of average molecular mass (*M*_m_) of control and treated samples ranged from 751 000 to 1 550 000 g/mol and from 932 000 to 1 790 000 g/mol, respectively. Ojima *et al*. (*13*) and Su *et al*. (*44*) found that the glucomannan flour treated with citric acid undergoes the degradation of glycoside bonds in the glucomannan molecules. This degradation is intensified by increasing the treatment temperature (*41*). The acid treatment led to grater destruction of the molecules during acid hydrolysis, resulting in short fragments of polysaccharide chains. The effect of NaCl could be explained by the strength of the chloride ion when interacting with water and its ability to precipitate polysaccharides. Hofmeister found that anions follow a certain order (SO_4_^2^ˉ>Fˉ>Clˉ>Brˉ>NO_3_ˉ>ClO_4_ˉ>Iˉ>SCNˉ) in their ability to precipitate polysaccharides. Anions on the left side of the series are called salting-out salts due to their strong interaction with water molecules, while right-side anions are called salting-in salts. NaCl has salting-out properties, so the ions of NaCl in the soaking solutions affect the interaction between the starch/glucomannan and water molecules. NaCl strengthens the polysaccharide-water molecular bond in the matrix of porang (*45*). The higher the temperature, the more Na^+^ and Cl^-^ ions diffuse into the matrix of porang slices. This reduces the leaching of polysaccharide molecules into the medium. At a temperature above 60 °C, it was observed that the temperature changed from the gel properties to the solution (*21*, *46*). It was suggested that higher glucomannan leaching at a soaking temperature of 85 °C resulted in a lower glucomannan content at this temperature. The lack of degradation of the glucomannan polymer chain resulted in a lower number of polymers with a certain molecular mass (*M*_i_), so the *M*_n_ values of the salt-treated samples were lower than of the acid-treated samples, which contributed to the decrease in their *M*_m_ values. In addition, Moreau *et al*. (*12*) observed that the addition of NaCl to starch triggered starch degradation at high temperatures and reduced the *M*_n_ and *M*_m_ values of starch.

The PDI value of the control samples, as shown in Table 2, is 1.35, while the acid- and salt-treated samples are between 1.23 and 1.34 and 1.16 and 1.24, respectively. The acid-treated samples showed a higher PDI value than the salt-treated samples, which increased with the soaking temperature. Generally, high PDI was attributed to the degradation of the polymer chains. This is thought to be due to the degradation of polysaccharide chains to shorter polysaccharide chains. The lower PDI of the salt-treated samples could be due to the rearrangement of polysaccharide chains, which increased with increasing temperature (*47*). Therefore, soaking in acid reduced the homogeneity of the molecular chains of porang flour and was enhanced by the increase in temperature, while soaking in salt solution increased the homogeneity.

### Thermal properties

**Fig. 3:**
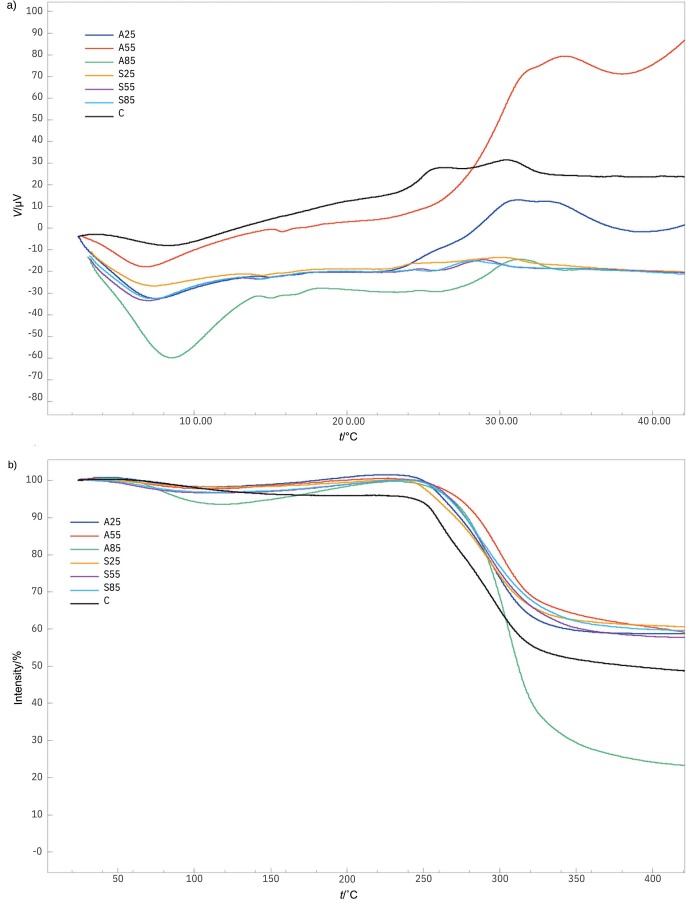
The curves of: a) differential thermal analysis (DTA), and b) thermogravimetric analysis (TGA) of porang flour obtained by soaking in acid and salt solution. C=control/untreated porang flour, A25, A55 and A85=soaked in 5 % citric acid at 25, 55 and 85 °C, respectively, S25, S55 and S85=soaked in 8 % sodium chloride at 25, 55 and 85 °C, respectively

The thermal properties of the control and treated samples are listed in Table 3. The curves of differential thermal analysis (DTA) and thermogravimetric analysis (TGA) are shown in Fig. 3a and Fig. 3b, respectively. The thermogram of the samples showed two different peaks, which is consistent with previous studies on glucomannan (*47*). The first peak observed in the samples occurred at around 100 °C and was identified as an endothermic peak. This peak was attributed to the removal of free or bound water from the samples. The last peak showed an exothermic peak in the temperature range of 235.13 to 342.75 °C (Table 3). This peak can be attributed to the breakdown reaction and chain fragmentation of the polysaccharides.

**Table 3:** Thermal properties of porang flour obtained by soaking in acid and salt solutions

**Treatment**	Thermal property				
	Mass loss/%	*t*_o_/°C	*t*_p_/°C	*t*_c_/°C	Δ*H*/(cal/g)
**C**	(41.3±1.3)	(246.4±0.7)	(304.24±0.00)	(320.0±0.1)	(137.9±11.9)
**A25**	(42.0±0.4)^d^	(272.5±0.4)^c^*	(310.66±0.00)^d^*	(351.0±6.5)^c^*	(224±20)^d^*
**A55**	(39.5±0.3)^c^	(316.29±0.00)^d^*	(342.75±0.00)^f^*	(364.5±0.4)^c^*	(159.3±5.2)^c^
**A85**	(67.5±0.8)^e^*	(275.0±0.1)^c^*	(312.51±0.00)^e^*	(332.58±0.04)^b^*	(81.3±4.1)^b^*
**S25**	(36.2±0.4)^ab^*	(235.1±1.8)^a^*	(300.3±1.6)^c^	(32217±1.0)^b^	(72.1±5.8)^b^*
**S55**	(35.3±0.2)^a^*	(272.3±6.1)^bc^*	(288.74±0.00)^b^*	(306.0±1.3)^a^*	(39.2±5.9)^a^*
**S85**	(36.8±0.9)^b^*	(264.2±5.6)^b^*	(281.56±0.00)^a^*	(307±12)^a^	(40.7±0.8)^a^*

Results are average value±standard deviation, *N*=3. Different letters in superscript in each column indicate that the values are statistically significantly different (p<0.05). *Statistically significant difference between the treated samples and the untreated sample according to Dunnett's test at p<0.05. C=control/untreated porang flour; A25, A55 and A85=soaked in 5 % citric acid at 25, 55 and 85 °C, respectively, S25, S55 and S85=soaked in 8 % sodium chloride at 25, 55 and 85 °C, respectively. *t*_o_, *t*_p_ and *t*_c_=onset, peak and endset temperature, respectively

The thermogravimetric curves in Fig. 3b also show two mass losses that correspond to their DTA curves. The first mass loss at the endothermic peak of around 100 °C was from 2 to 6 % (the mass loss around 100 °C is mainly due to water loss). The control and acid-treated samples lost more mass than the salt-treated samples, suggesting their lower water-holding capacity. The mass loss at the exothermic peak of the control and treated samples was 41.32 % and ranged from 35.34 to 67.49 %, respectively, as shown in Table 3. The mass loss of the control sample was attributed to many organic components such as glucose, protein and polysaccharides that were present in the control sample (*21*). The salt-treated samples had a lower mass loss than the acid-treated samples. The acid treatment shortened the molecular chains so that they could decompose more easily. Increasing the soaking temperature led to an increase in mass loss, especially at a soaking temperature of 85 °C, which was consistent with the molecular mass results. Therefore, the soaking in salt solution improved the thermal properties of porang flour better than soaking in acid solution based on the mass loss.

Table 3 shows the thermal properties of the decomposition process of the samples (exothermic peak). The onset (*t*_o_), peak (*t*_p_) and endset (*t*_c_) temperatures of the samples ranged from 235.1 to 316.29 °C, from 281.56 to 342.75 °C and from 306.0 to 364.5 °C, respectively. The increase in *t*_o_ and *t*_c_ of the treated sample shows that they are more thermally stable than the control sample. This is related to the removal of some impurities (Ca-oxalate and starch) due to the soaking treatment. In addition, salt-treated samples showed lower thermal properties (*t*_c_, *t*_p_ and Δ*H*) than the acid-treated samples. The addition of NaCl observed by Moreau *et al*. (*12*) reduced the crystallinity of the starch granules. The low crystallinity of the sample reduces the exothermic peak temperature of the polysaccharide (*48*).

### Rheological properties

**Fig. 4:**
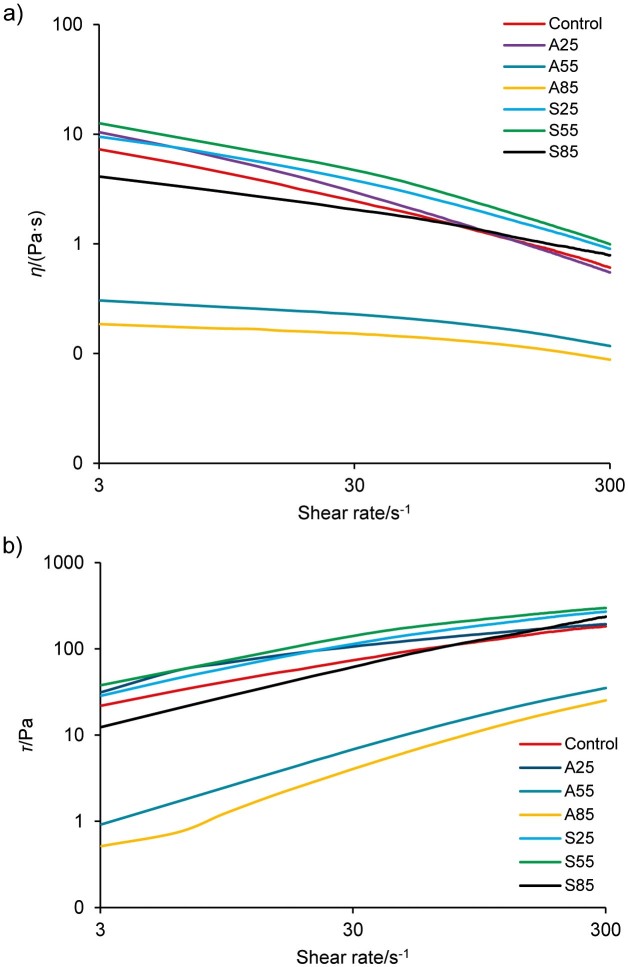
Determination of: a) apparent viscosity (*η*), and b) shear stress (*τ*) of porang flour obtained by soaking in acid and salt solutions. C=control/untreated porang flour, A25, A55 and A85=soaked in 5 % citric acid at 25, 55 and 85 °C, respectively, S25, S55 and S85=soaked in 8 % sodium chloride at 25, 55 and 85 °C, respectively

Viscosity is an important property of hydrogel materials such as glucomannan. The apparent viscosity and shear stress of control and treated samples at different shear rates are shown in Fig. 4. The bar chart in Fig. 4a shows the apparent viscosity at a constant shear rate of 12 rpm. Fig. 4a also shows that the apparent viscosity decreased with increasing shear rate and demonstrates typical shear thinning behaviour. Fig. 4b shows that the shear stress increased with increasing shear rate for all samples. This behaviour can be explained by the breakdown of the network of entangled polysaccharide molecules during the shearing process. At a low shear rate, the viscosity does not change much with increasing shear rate, and the force required to break this entanglement is also low. At high shear rates, however, with the increase in shear rates, the rate at which the polysaccharide molecules disentangle is greater than their re-entanglement rate. Therefore, the intermolecular resistance to flow decreases and the viscosity becomes lower (*21*). The high disentanglement rate of the polysaccharide molecules requires higher shear stress.

Fig. 4a shows that the viscosity of the salt-treated samples is higher than that of the acid-treated samples. In the Hofmeister series, NaCl can have the strength of salting out in the middle, depending on the concentration. The increase in viscosity can be attributed to the conformational change of glucomannan molecule triggered by NaCl. The electrostatic effect of the NaCl ions increases the expansion of glucomannan molecules, leading to a subsequent increase in the viscosity of the glucomannan solution (*49*, *50*). Zhang *et al*. (*50*) also reported that the viscosity of the starch-glucomannan mixture increases at NaCl concentrations higher than 0.1 mol/L.

Moreover, higher soaking temperatures resulted in samples with lower viscosity values, with the exception of sample S55. The apparent viscosity of the samples soaked in acid solution at temperatures of 55 and 85 °C changed. They tended to show a constant value regardless of the shear rate and behaved like a Newtonian fluid. Soaking the porang tubers in acid solution at higher temperatures may destroy polysaccharide molecules during acid hydrolysis, resulting in more short-chain polysaccharides, as shown in Table 2. Furthermore, increasing the temperature of NaCl solution increased the viscosity of the samples. However, at the soaking temperature of 85 °C, the viscosity of the samples decreased. This is related to the glucomannan and ash mass fraction in sample S85, which was lower than in samples S25 and S55 (indicating the amount of NaCl deposited) in Table 1. Therefore, soaking the porang tubers in a salt solution, especially at 55 °C (S55), resulted in better rheological properties than those of an acid solution.

### Morphology of porang flour

**Fig. 5:**
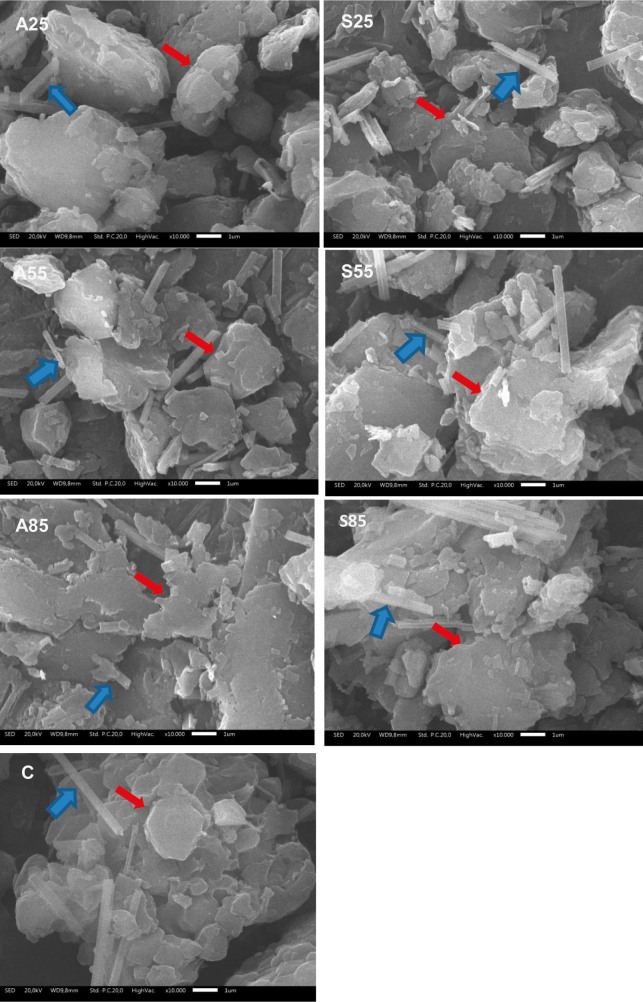
Morphology of porang flour obtained by soaking in acid and salt solutions. C=control/untreated porang flour, A25, A55 and A85=soaked in 5 % citric acid at 25, 55 and 85 °C, respectively, S25, S55 and S85=soaked in 8 % sodium chloride at 25, 55 and 85 °C, respectively

The morphology of porang flour is shown in Fig. 5. Native porang flour (C) has a smooth surface, while the treated samples have a slightly rough surface, as shown by the red arrow in Fig. 5. The acid and salt treatments caused damage, as shown by the irregular surface of the porang flour. The acid treatment caused the granule surface to become irregular and eroded due to their corrosion properties (*51*, *52*). Treatment with acid at a high temperature (85 °C) showed that the surface of the samples became rougher, as shown in Fig. 5. Su *et al.* (*44*) also observed that the surface structure of the glucomannan particles had a slightly roughened surface and a small indentation caused by the addition of citric acid at 60 °C, probably due to citric acid absorption on glucomannan particles.

Further analysis showed that the porang flour samples contained a lot of needle-shaped or raphide Ca-oxalate crystals, as shown by the blue arrow in Fig. 5. The treatment with acid or salt shortened the needle-shaped crystals of Ca-oxalate compared to the control, resulting in a reduction of Ca-oxalate crystals in the porang flour. Soaking the tubers of *Xanthosoma sagittifolium* in acetic acid caused the Ca-oxalate crystals to separate microscopically and reduce in number (*23*). In Fig. 5, an increase in the soaking temperature also caused a reduction in Ca-oxalate crystals. This was confirmed by a decrease in the Ca-oxalate mass fraction in the porang flour (Table 1). Similar results were obtained by Kumoro *et al*. (*53*), who indicated that thermal deterioration, the synergistic effect of leaching and the thermally labile properties of Ca-oxalate were the causes of the reduction of Ca-oxalate crystals during high-temperature treatment in salt solution. The treatment at high temperature in acid and salt solutions causes granule damage, which facilitates the removal of Ca-oxalate crystals in porang flour.

## CONCLUSIONS

Soaking of porang slices in acid and salt solutions at different temperatures (25, 55 and 85 °C) affects the properties of porang flour. The properties of porang flour soaked in salt were better than those of the flour soaked in acid, such as lower Ca-oxalate, starch mass fraction, thermal properties and higher glucomannan mass fraction and viscosity. Salt treatment at 55 °C is considered to be the most effective, as it leads to a low Ca-oxalate content and the highest glucomannan content and viscosity. In addition, this treatment resulted in the lowest polydispersity index and mass loss (thermal properties). Based on morphological observations, the shortened needle-shaped Ca-oxalate crystals indicated a reduction of the Ca-oxalate crystals.

## References

[1] Yanuriati A Marseno DW. Rochmadi, Harmayani E. Characteristics of glucomannan isolated from fresh tuber of porang (*Amorphophallus muelleri* Blume). Carbohydr Polym. 2017;156:56–63. 10.1016/j.carbpol.2016.08.080 27842852

[2] Zainuri S Basuki E Handayani BR Sulastri Y. Paramartha DNA, Anggraini MD. Optimization process to increase the quality of Lombok porang flour. IOP Conf Ser Earth Environ Sci. 2021;913(1):012037. 10.1088/1755-1315/913/1/012037

[3] Shah SWA Jahagir M Qaisar M Khan SA Mahmood T Saeed M Storage stability of kinnow fruit (*Citrus reticulata*) as affected by CMC and guar gum-based silver nanoparticle coatings. Molecules. 2015;20(12):22645–61. 10.3390/molecules201219870 26694344 PMC6332021

[4] Chairiyah N Harijati N Mastuti R. The dynamic of calcium oxalate (Caox) in porang corms (*Amorphophallus muelleri* Blume) at different harvest time. J Trop Life Sci. 2021;11(1):33–44. 10.11594/jtls.11.01.05

[5] Wardani RK, Arifiyana D. The effect of soaking time and temperature of acetic acid solution to the de-crease of calcium oxalate levels in porang tubers. 1^st^ International Conference Eco-Innovation in Science, Engineering, and Technology. NST Proceedings; 2020. pp. 145–9. 10.11594/nstp.2020.0522

[6] Hermanto MB Widjanarko SB Suprapto W Suryanto A. The design and performance of continuous porang (*Amorphophallus muelleri* Blume) flour mills. Int J Adv Sci Eng Inf Technol. 2019;9(6):2021–7. 10.18517/ijaseit.9.6.6396

[7] Adejumo BA Asema JK. Effect of hydrothermal treatment on selected properties of cocoyam corm (*Colocasia esculenta*) flour. Indones Food Sci Technol J. 2022;5(2):44–8. 10.22437/ifstj.v5i2.14554

[8] Medoua GN Mbome IL Egbe TA Mbofung CMF. Salts soaking treatment for improving the textural and functional properties of trifoliate yam (*Dioscorea dumetorum*) hardened tubers. J Food Sci. 2007;72(8):E464–9. 10.1111/j.1750-3841.2007.00511.x 17995606

[9] Desalegn Melese A Keyata EO. Impacts of pretreatment techniques on the quality of tuber flours. Sci World J. 2022;2022:9323694. 10.1155/2022/9323694 35795013 PMC9252694

[10] Grancher D Soudy ID Delatour P. Effects of traditional soaking on the nutritional profile of taro flour (*Colocasia esculenta* L. Schott) produced in Chad. Rev Med Vet (Toulouse). 2010;161(1):37–42.

[11] Ugwuanyi CA Asogwa IS Ani JC. The effect of boiling and soaking time on the chemical and functional properties of wild bitter yam. Int J Food Sci Nutr. 2020;5(4):63–8.

[12] Moreau L Bindzus W Hill S. Influence of salts on starch degradation: Part I - Sodium chloride. Starch/Stärke. 2011;63(11):669–75. 10.1002/star.201100031

[13] Ojima R Makabe T Prawitwong P Takahashi R Takigami M Takigami S. Rheological property of hydrolyzed konjac glucomannan. Trans Mater Res Soc Jpn. 2009;34(3):477–80. 10.14723/tmrsj.34.477

[14] Sarifudin A Ratnawati L Indrianti N Ekafitri R Sholichah E Afifah N. Evaluation of some analytical methods for determination of calcium oxalate in *Amorphophallus muelleri* flour. Food Sci Technol. 2022;42:09522. 10.1590/fst.09522

[15] Iwuoha CI Kalu FA. Calcium oxalate and physico-chemical properties of cocoyam (*Colocasia esculenta* and *Xanthosoma sagittifolium*) tuber flours as affected by processing. Food Chem. 1995;54(1):61–6. 10.1016/0308-8146(95)92663-5

[16] Official Method AOAC. 923.03. Ash of flour. Rockville, MD, USA: AOAC International; 2005.

[17] Sudarmaji S, Haryono B, Suhardi. Prosedur analisis untuk bahan makanan (Food and agricultural material analysis). Liberty, Yogyakarta, Indonesia; 1997 (in Indonesian).

[18] Perez CM Juliano BO. Modification of the simplified amylose test for milled rice. Stärke. 1978;30(12):424–6. 10.1002/star.19780301206

[19] Peiying L, Shenglin Z, Guohua Z, Yan C, Huaxue O, Mei H. Konjac flour. Professional Standard of the People's Republic of China. Bejing, PR China: Ministry of the People’s Republic of China; 2002. Available from: http://www.konjacfoods.com/pdf/NY494-cn.pdf.

[20] Sholichah E Purwono B Murdiati A Syoufian A Sarifudin A. Extraction of glucomannan from porang (*Amorphophallus muelleri* Blume) with freeze-thaw cycles pre-treatment. Food Sci Technol. 2023;43(5):1423. 10.5327/fst.1423

[21] Xu W Wang S Ye T Jin W Liu J Lei J A simple and feasible approach to purify konjac glucomannan from konjac flour - Temperature effect. Food Chem. 2014;158:171–6. 10.1016/j.foodchem.2014.02.093 24731328

[22] IBM SPSS Statistics for Windows, v. 26, IBM Corp. Armonk, NY, USA; 2019. Available from: https://www.ibm.com/id-id/products/spss-statistics/gradpack.

[23] Agustin R Estiasih T Wardani A. Decrease of oxalate on construction process of new cocoyam (*Xanthosoma sagittifolium*) in various concentration of acetic acid. J Teknol Pertan. 2017;18(3):191–200. 10.21776/ub.jtp.2017.018.03.19

[24] Toshima S. Beryllium, magnesium, calcium, strontium, barium and radium. In: Greenwood NN, Earnshaw A, editors. Chemistry of the elements, vol. 34. Oxford, UK: Butterworth-Heinemann; 1997. pp. 107–38.

[25] Trithavisup K Charoenrein S. Influence of acid treatment on physicochemical properties of aged rice flour. Int J Food Prop. 2016;19(9):2074–86. 10.1080/10942912.2015.1104510

[26] Ayele E Urga K Chandravanshi BS. Effect of cooking temperature on mineral content and anti-nutritional factors of yam and taro grown in southern Ethiopia. Int J Food Eng. 2015;11(3):371–82. 10.1515/ijfe-2014-0264

[27] Bahlawan ZAS Damayanti A. Megawati, Cahyari K, Andriani N, Hapsari RA. Study of glucomannan extraction with hydrochloric acid catalyst and alcohol solvent based on porang tuber flour (*Amorphophallus oncophyllus*). IOP Conf Ser Earth Environ Sci. 2021;700(1):012069. 10.1088/1755-1315/700/1/012069

[28] Salehi F Kashaninejad M. Kinetics and thermodynamics of gum extraction from wild sage seed. Int J Food Eng. 2014;10(4):625–32. 10.1515/ijfe-2014-0079

[29] Rosida DF, Yuliani R, Djajati S. Modification of *Colocasia esculenta* starch with acetylation process. NST Proceedings. 2020;2019:369–78. 10.11594/nstp.2019.0453

[30] Luo H Liang D Liu Q Zheng Y Shen H Li W. Investigation of the role of sodium chloride on wheat starch multi-structure, physicochemical and digestibility properties during X-ray irradiation. Food Chem. 2024;447:139012. 10.1016/j.foodchem.2024.139012 38492296

[31] Olsson E Menzel C Johansson C Andersson R Koch K Järnström L. The effect of pH on hydrolysis, cross-linking and barrier properties of starch barriers containing citric acid. Carbohydr Polym. 2013;98(2):1505–13. 10.1016/j.carbpol.2013.07.040 24053833

[32] Sumardiono S Rakhmawati RB Pudjihastuti I. Physicochemical and rheological properties of sago (*Metroxylon sagu*) starch modified with lactic acid hydrolysis and UV rotary drying. ASEAN J Chem Eng. 2018;18(2):41–53.

[33] Donoso-Bravo A Retamal C Carballa M Ruiz-Filippi G Chamy R. Influence of temperature on the hydrolysis, acidogenesis and methanogenesis in mesophilic anaerobic digestion: Parameter identification and modeling application. Water Sci Technol. 2009;60(1):9–17. 10.2166/wst.2009.316 19587397

[34] Mir SA Bosco SJD Bashir M Shah MA Mir MM. Physicochemical and structural properties of starches isolated from corn cultivars grown in Indian temperate climate. Int J Food Prop. 2017;20(4):821–32. 10.1080/10942912.2016.1184274

[35] Wang S Zhou B Wang Y Li B. Preparation and characterization of konjac glucomannan microcrystals through acid hydrolysis. Food Res Int. 2015;67:111–6. 10.1016/j.foodres.2014.11.008

[36] Akubor PI. Effect of ascorbic acid and citric acid treatments on the functional and sensory properties of yam flour. Int J Agric Policy Res. 2013;1(4):103–8.

[37] Wibowo C Haryanti P. Erminawati, Wicaksono R. Effect of blanching method and soaking solution on the properties of potato flour produced from variety Granola. IOP Conf Ser Earth Environ Sci. 2019;255(1):012021. 10.1088/1755-1315/260/1/012021

[38] Wang S Copeland L. Molecular disassembly of starch granules during gelatinization and its effect on starch digestibility: A review. Food Funct. 2013;4(11):1564–80. 10.1039/c3fo60258c 24096569

[39] Widjanarko SB Nugroho A Estiasih T. Functional interaction components of protein isolates and glucomannan in food bars by FTIR and SEM studies. Afr J Food Sci. 2011;5(1):12–21.

[40] Dolas KA Ranveer RC Tapre AR Nandane AS Sahoo AK. Effect of starch modification on physico-chemical, functional and structural characterization of cassava starch (*Manihot esculenta* Crantz). Food Res. 2020;4(4):1265–71. 10.26656/fr.2017.4(4).075

[41] Azhar B Gunawan S Setyadi ERF Majidah L Taufany F Atmaja L Purification and separation of glucomannan from porang tuber flour (*Amorphophallus muelleri*) using microwave assisted extraction as an innovative gelatine substituent. Heliyon. 2023;9(11):e21972. 10.1016/j.heliyon.2023.e21972 38034783 PMC10682115

[42] Derrick MR, Stulik D, Landry JM. Infrared spectroscopy in conservation science. Los Angeles, CA, USA: Getty Conservation Institute; 1999.

[43] Khuwijitjaru P Koomyart I Kobayashi T Adachi S. Hydrolysis of konjac flour under subcritical water conditions. Warasan Khana Witthayasat Maha Witthayalai Chiang Mai. 2017;44(3):988–92.

[44] Su Y Zhang M Chang C Li J Sun Y Cai Y The effect of citric-acid treatment on the physicochemical and gel properties of konjac glucomannan from *Amorphophallus bulbifer.* Int J Biol Macromol. 2022;216:95–104. 10.1016/j.ijbiomac.2022.06.199 35793743

[45] da Silva DF Ogawa CYL Sato F Neto AM Larsen FH Matumoto-Pintro PT. Chemical and physical characterization of Konjac glucomannan-based powders by FTIR and ^13^C MAS NMR. Powder Technol. 2020;361:610–6. 10.1016/j.powtec.2019.11.071

[46] Lins AC de A. Cavalcanti DT de B, Azoubel PM, Mélo E de A, Maciel MIS. Effect of hydrocolloids on the physicochemical characteristics of yellow mombin structured fruit. Food Sci Technol. 2014;34(3):456–63. 10.1590/1678-457x.6348

[47] Kurt A Kahyaoglu T. Purification of glucomannan from salep: Part 2. Structural characterization. Carbohydr Polym. 2017;169:406–16. 10.1016/j.carbpol.2017.04.052 28504162

[48] Li J Ye T Wu X Chen J Wang S Lin L Preparation and characterization of heterogeneous deacetylated konjac glucomannan. Food Hydrocoll. 2014;40:9–15. 10.1016/j.foodhyd.2014.02.001

[49] Yin W Zhang H Huang L Nishinari K. Effects of the lyotropic series salts on the gelation of konjac glucomannan in aqueous solutions. Carbohydr Polym. 2008;74(1):68–78. 10.1016/j.carbpol.2008.01.016

[50] Zhang F Liu M Mo F Zhang M Zheng J. Effects of acid and salt solutions on the pasting, rheology and texture of lotus root starch-konjac glucomannan mixture. Polymers (Basel). 2017;9(12):695. 10.3390/polym9120695 30965995 PMC6418512

[51] Anyasi TA Jideani AIO Mchau GRA. Effects of organic acid pretreatment on microstructure, functional and thermal properties of unripe banana flour. J Food Meas Charact. 2017;11(1):99–110. 10.1007/s11694-016-9376-2

[52] Palma-Rodriguez HM Agama-Acevedo E Mendez-Montealvo G Gonzalez-Soto RA Vernon-Carter EJ Bello-Pérez LA. Effect of acid treatment on the physicochemical and structural characteristics of starches from different botanical sources. Starch/Stärke. 2012;64(2):115–25. 10.1002/star.201100081

[53] Kumoro AC Budiyati CS Retnowati DS. Calcium oxalate reduction during soaking of giant taro (*Alocasia macrorrhiza* (L.) Schott) corm chips in sodium bicarbonate solution. Int Food Res J. 2014;21(4):1583–8.

